# TRUNCATULIX – a data warehouse for the legume community

**DOI:** 10.1186/1471-2229-9-19

**Published:** 2009-02-11

**Authors:** Kolja Henckel, Kai J Runte, Thomas Bekel, Michael Dondrup, Tobias Jakobi, Helge Küster, Alexander Goesmann

**Affiliations:** 1 Bioinformatics Resource Facility, Center for Biotechnology, Bielefeld University, Bielefeld, Germany; 2 International Graduate School in Bioinformatics and Genome Research, Bielefeld University, Bielefeld, Germany; 3 Technical Faculty, Bielefeld University, Bielefeld, Germany; 4 Computational Genomics, Center for Biotechnology, Bielefeld University, Bielefeld, Germany; 5 Faculty for Biology and Genetics, Bielefeld University, Bielefeld, Germany; 6 Genomics of Legume Plants, Institute for Genome Research and Systems Biology, Center for Biotechnology, Bielefeld University, Bielefeld, Germany; 7 Unit IV – Plant Genomics, Institute for Plant Genetics, Leibniz Universität Hannover, Germany

## Abstract

**Background:**

Databases for either sequence, annotation, or microarray experiments data are extremely beneficial to the research community, as they centrally gather information from experiments performed by different scientists. However, data from different sources develop their full capacities only when combined. The idea of a data warehouse directly adresses this problem and solves it by integrating all required data into one single database – hence there are already many data warehouses available to genetics. For the model legume *Medicago truncatula*, there is currently no such single data warehouse that integrates all freely available gene sequences, the corresponding gene expression data, and annotation information. Thus, we created the data warehouse TRUNCATULIX, an integrative database of *Medicago truncatula *sequence and expression data.

**Results:**

The TRUNCATULIX data warehouse integrates five public databases for gene sequences, and gene annotations, as well as a database for microarray expression data covering raw data, normalized datasets, and complete expression profiling experiments. It can be accessed via an AJAX-based web interface using a standard web browser. For the first time, users can now quickly search for specific genes and gene expression data in a huge database based on high-quality annotations. The results can be exported as Excel, HTML, or as csv files for further usage.

**Conclusion:**

The integration of sequence, annotation, and gene expression data from several *Medicago truncatula *databases in TRUNCATULIX provides the legume community with access to data and data mining capability not previously available. TRUNCATULIX is freely available at .

## Background

*Medicago truncatula *is a model plant for studying legume biology. Legumes are mainly characterized by their ability to interact with beneficial microbial organisms, leading to the formation of nitrogen-fixing root nodules and to phosphate-acquiring arbuscular mycorriza. Various international research projects are investigating these different symbioses of *Medicago truncatula*. The arbuscular mycorrhiza (AM) interaction between the host root and the fungal partner is an interesting field of research because more than 80% of land plants depend on an efficient AM for the uptake of nutrients, primarily phosphate. Apart from AM, *Medicago truncatula *is capable of entering a nitrogen-fixing symbiosis with the soil bacterium *Sinorhizobium meliloti*. The capacity for symbiotic nitrogen fixation allows legumes such as *Medicago truncatula *to grow on nitrogen-depleted soils and to develop protein-rich seeds, which are properties exploited in sustainable agriculture [1-5].

In recent years, more and more databases for the storage of microarray expression data (Arrayexpress [6], PEPR [7], The Stanford MicroArray Database [8], PlexDB [9]), and data from different sequencing projects (EST sequencing(dbEST [10]), BAC sequencing (GenMapDB [11]), ultrafast sequencing (Short Read Archive [12])), have been developed to store the exponentially growing amount of data. However, scientists have to search for the specific information in each and every database separately.

Directly adressing this issue, data warehouses, specially designed databases, offer an approach to store different aspects of a certain data object in an optimized data schema. This provides fast data access and enables return of query results in minimal time [13,14].

To overcome the problem of distributed data sources in the field of *Medicago truncatula *research, we created TRUNCATULIX, a data warehouse storing sequence data, annotations, and expression experiments of the model legume *Medicago truncatula*.

## Construction and content

The construction of the TRUNCATULIX data warehouse is divided in three main parts: 1) the database schema, 2) the data integration, 3) and the source data. The following sections outline these three aspects in detail.

### Database schema

**Figure 1:**
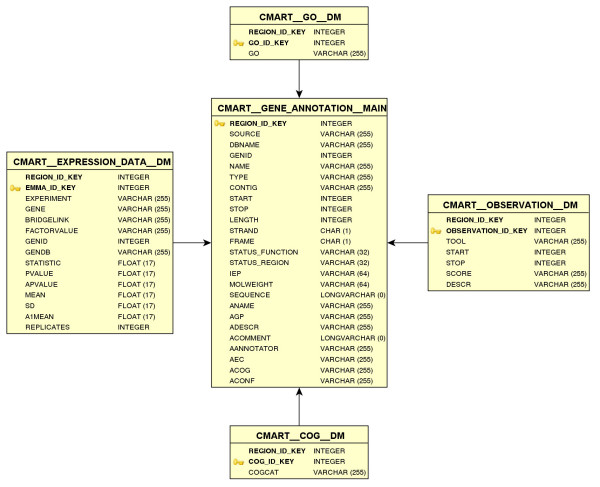
**The TRUNCATULIX database schema**. The TRUNCATULIX database schema. The main table stores the sequence data. All other information is stored in different tables refering to the main table.

The intention of the TRUNCATULIX data warehouse is to store information on gene sequences, functional annotations, and expression data. Thus, we created a relational data schema (see Figure 1), containing all these aspects. The different tables that store the information are linked via unique keys.

The TRUNCATULIX data warehouse is based on the IGetDB data warehouse engine [15] using Java [16]. It uses MySQL [17] as the database management system and is based on the Biomart API [18], while providing additional functionality such as full-text searches and direct access to SQL-functions (e.g. SUM, AVG, ROUND). The primary gene information such as sequence data, start codon, stop codon, length of the encoded open reading frame, name, or gene_id are stored in the main table, whereas data such as gene expression values from different experiments, GO numbers, or KOG categories are stored in extra tables refering to the main table.

### Data integration

High throughput technologies yield vast amounts of data. However, in order to investigate, for example, regulatory pathways, further standard genetics approaches are yet most effective. High throughput data provide an excellent means to reduce the number of possible mutation targets. Such data is found, in general, in different sources and the screening of (most likely) thousands of candidates is, when performed manually, a rather tedious task. On the other hand, if the data was integrated and preprocessed for querying, such a task can be performed in a matter of minutes. As has been shown recently, such data integration strategies saves both time and money [19].

For the integration and import of data we use the extract, transform and load (ETL) approach commonly used in data warehousing [20]. Sequence and annotation data are extracted from SAMS (cf. next section), and are subsequently transformed and loaded into the TRUNCATULIX database. Expression data is exported from EMMA (cf. Section "Expression data") via an export script that can be initiated by a human curator within the EMMA web interface. During the transformation step, the expression data are linked to the sequence data. This enables the user of TRUNCATULIX to conveniently search across annotation and expression data.

### Data sources

#### Sequence data

• *Medicago truncatula *Gene Index 8.0

The Institute for Genomic Research (TIGR – J. Craig Venter Institute since October 2006) clustered and assembled 226,923 high-quality ESTs from over 60 different *Medicago truncatula *EST-libraries sequenced in laboratories all over the world. Using the clustering software *tgicl *[21], the *Medicago truncatula *Gene Index (MtGI, hosted at the Dana-Farber Cancer Institute – DFCI), was built. The MtGI 8.0 contains 18,612 Tentative Consensus sequences (TCs) and 18,238 singletons (Jan. 2005) [22]. The sequences were imported into the Sequence Analysis and Management System (SAMS) [23], an annotation software created at the Center for Biotechnology (CeBiTec) in Bielefeld. The SAMS system contains an automatic annotation pipeline (Metanor), which runs several bioinformatics tools for gene annotation (Blast, Interpro, TMHMM) [24-26]. A high quality consensus annotation is created, covering EC numbers [27], KEGG functions [27], GO numbers [28], KOG numbers [29], putative gene functions, and gene names.

• *Medicago truncatula *Gene Index 9.0

Recently, the J. Craig Venter Institute released a new version of the *Medicago truncatula *Gene Index, now covering over 70 EST-libraries. The assembly of the 259,642 ESTs led to 29,273 TCs, while 26,696 ESTs remained as singletons. In addition to the previous Gene Index 8.0, TIGR used 25,600 mature transcripts (ETs) from the qcGene Database [30] for the EST assembly, whereof 11,494 ETs remained as singletons. The new sequences were downloaded from the DFCI websites and imported into SAMS, where a complete automatic annotation was performed.

• *Medicago *genome project

The *Medicago *Genome Sequence Consortium (MGSC) sequenced the *Medicago truncatula *genome using a classical BAC sequencing approach [31,32]. Starting in 2005, they released a assembly of the sequences in October 2007 (release 2.0). This release contains 38,759 coding sequences (CDS) and the same number of translated protein sequences. The CDS's were downloaded from the project website and afterwards imported into SAMS. Using SAMS, a complete automatic annotation was performed.

• Affymetrix *Medicago *GeneChip^® ^probes

Affymetrix [33] offers a GeneChip^® ^microarray holding probes primarily for genes of *Medicago truncatula*, but also for the related legume *Medicago sativa *and their symbiontic *Sinorhizobium meliloti*. The sequences used by Affymetrix to construct the *Medicago *Genome GeneChip^® ^were downloaded from the Affymetrix website and imported into SAMS. That way, 61,103 sequences containing the Affymetrix annotations were integrated into SAMS and were automatically re-annotated using the Metanor pipeline.

• *Medicago truncatula *454 sequencing project

In 2006, Cheung *et al*. used the pyrosequencing approach to generate 292,465 cDNA reads of *Medicago truncatula *using a GS20 sequencer [34]. The reads were assembled forming 3,619 sequences. These sequences were downloaded from the project website and imported into SAMS. Using SAMS, a complete automatic annotation was performed.

Using Blast homology search, the sequences of all five projects were compared against the sequences of the other projects (one-by-one). This way, the sequences corresponding to each other in the different datasets could be found (using an e-value cutoff of *e*^-5^). The complete sequence and annotation data from all five projects was integrated into the TRUNCATULIX data warehouse. Table 1 presents the number of sequences imported into TRUNCATULIX from the five projects, giving additional information about the automatic annotation.

**Table 1:** Sequence data integrated into TRUNCATULIX.

**Project**	**Sequences**	**EC numbers**	**KOG numbers**	**GO numbers**
MtGI 8.0 TCs & Singletons	36,878	6,174 (16.74%)	12,746 (34.56%)	10,268 (27.84%)
MtGI 9.0 TCs & Singletons	67,463	11,253 (16.68%)	20,570 (30.49%)	19,008 (28.18%)
Mt Genome 2.0	38,759	5,938 (15.32%)	3,434 (8.85%)	10,444 (26.95%)
Affymetrix Medicago				
GeneChip^® ^probes	61,103	12,044 (19.71%)	18,731 (30.65%)	19,775 (32.36%)
Medicago 454 sequencing project	3,619	911 (25.17%)	1,798 (49.68%)	519 (14.34%)

**Total**	**207,822**	**36,320 (17.48%)**	**57,278 (27.56%)**	**60,014 (28.88%)**

The table shows the number of sequences integrated in TRUNCATULIX via the SAMS software. The annotation information was calculated using various bioinformatic tools.

#### Expression data

• Oligo-microarray expression data

In recent years, almost 1,000 oligo-microarrays studying *Medicago truncatula *gene expression in different conditions were hybridized in the framework of various international projects [35]. These microarrays used two chip layouts designated Mt16kOLI1 [5] and Mt16kOLI1Plus [36] (Arrayexpress ID: A-MEXP-85/A-MEXP-138). These arrays are associated to more than 50 different expression profiling experiments that were analyzed via the EMMA [37,38] software. EMMA is a tool that analyses microarray expression data and stores it in a MIAME compliant way [39]. It is also MAGE compliant [40] and the analysis pipelines used are well documented and evaluated. EMMA supports various image analyses software, such as ImaGene [41] and GenePix [42]. Within EMMA it is possible to group different mircoarrays into experiments. The user can decide to filter the differentially expressed genes using different significance tests. Some of the results of these analyses can be found in [1-3,35]. Table 2 lists the currently available *Medicago truncatula *oligo-microarray experiments, as well as the number of microarrays and the number of transformed datasets integrated into TRUNCATULIX via EMMA. Some of the integrated data is already published in [43-45], some more is unpublished data by H. Küster(*), M. Hahn, N. Hohnjec, H. Küster(**), D. Hinse, A. Becker, H. Küster(***), C. Hogekamp, H. Küster(****) and F. Frugier(*****). More microarray experiments will be integrated as soon as the researchers in charge have approved their integration into the data warehouse.

**Table 2:** Microarray expression data imported from EMMA into TRUNCATULIX.

**Experiment**	**Number of microarrays**	**Number of transformed datasets**
Nitrogen-fixing root nodules in Medicago truncatula*	4	10
Nod-Factor response in *Medicago truncatula *roots [43]	9	13
Root endosymbiosis in *Medicago truncatula *[5]	10	23
Uromyces pathogenesis in Medicago truncatula**	3	4
AHL treatment of Medicago truncatula roots***	11	17
LMW EPS I treatment of *Medicago truncatula *roots I****	6	8
LMW EPS I treatment of *Medicago truncatula *roots II****	6	8
LMW EPS I treatment of *Medicago truncatula *roots III****	24	32
Nod-factor treatment of *Medicago truncatula *roots I****	6	8
Nod-factor treatment of *Medicago truncatula *roots II****	18	24
Seed development in *Medicago truncatula *[44]	22	51
Early Salt Stress in *Medicago truncatula *[45]	4	5
Cold stress in Medicago truncatula*****	8	11
*Medicago truncatula *wild type roots vs. TN1_11 mutant roots after 1 h of salt stress*****	16	20
Response to phosphate in *Medicago truncatula *roots [5]	3	4

**Total**	**150**	**248**

The table shows the amount of microarray expression data integrated into TRUNCATULIX via EMMA. Currently there are 15 different experiments with a total of 150 microarrays (248 transformed datasets) integrated. Some of the integrated data is unpublised: H. Küster(*), M. Hahn, N. Hohnjec, H. Küster(**), D. Hinse, A. Becker, H. Küster(***), C. Hogekamp, H. Küster(****) and F. Frugier(*****). Abbreviations: AHL: acetylhomoserine lactone, LMW: low molecular weight, EPS: exopolysaccharide, Nod-factor: Nodulation factor

• GeneChips^® ^expression data

In 2008, Benedito *et al*. hybridized more than 50 Affymetrix *Medicago truncatula *GeneChip arays^® ^[46], addressing three major topics: mature organs covering the whole plant, nodule development, and seed development. For each of these topics, four to eight experiments were perfomed in three replicates each (see Table 3). The expression data of the GeneChip arrays was downloaded and directly migrated into the TRUNCATULIX data warehouse.

**Table 3:** GeneChip data integrated into TRUNCATULIX.

**Experiment**	**Number of GeneChip arrays**
**Mature organs series**	**24**

Leaf: 4-week old trifolia were harvest without their petioles (but with their petiolule) [46]	3
Petiole: Petioles from 4-week old plant [46]	3
Stem: Stems of 4-week old plants (without vegetative buds) [46]	3
Vegetative Bud: Vegetative buds of 4-week old plants [46]	3
Root: 4-week old non-inoculated roots [46]	3
Nodule: Nodules from 4-week old plants [46]	3
Flower: Fully open flowers were harvest at the day of anthesis [46]	3
Pod: Mix of small, medium and physiologically mature pods [46]	3

**Nodulation development series**	**12**

Root-0d: Roots at 0 dpi (control for nodule developmental series) [46]	3
Nod4d: Nodules at 4 dpi (root lumps with residual roots) [46]	3
Nod10d: Developing nodules at 10 dpi [46]	3
Nod14d: Mature nodules at 14 dpi [46]	3

**Seed development series**	**18**

Seed10d: Developing seeds at early embryogenesis – 10 dap [46]	3
Seed12d: Developing seeds at 12 dap (transition between embryogenesis and seed filling) [46]	3
Seed16d: Developing seeds at 16 dap (accumulation of storage proteins) [46]	3
Seed20d: Developing seeds at 20 dap (seed filling) [46]	3
Seed24d: Developing seeds at 24 dap (maturation phase) [46]	3
Seed36d: Developing seeds at 36 dap (physiologically mature seeds, desiccation) [46]	3

**Total**	**54**

The table shows the experiments and number of GeneChip arrays^® ^directly imported into TRUNCATULIX. The three experiments address major topics: Mature organs covering the whole plant, nodulation development, and seed development.

The integrated data (sequence and expression data) is curated before it is imported into TRUNCATULIX. No user is allowed to import any datasets, this is reserved to the curators, but can be done upon request. In case of an update of one of the source databases the integrated data are updated by the curators.

## Utility and Discussion

TRUNCATULIX is a relational, integrated database of sequence data, annotation information and microarray expression data, which is specially created to store data of the model legume *Medicago truncatula*.

### Web interface

The interface for the TRUNCATULIX data warehouse can be accessed via a web browser. It provides a user-friendly interface built with Echo2 [47].

### Case study

Consider a query for genes concerning GRAS transcription factors, suggesting that these genes are activated during nodulation [48,49]. As an example, we search for genes annotated as GRAS transcription factors in microarray experiments covering nodulation with three or more replicates.

**Figure 2:**
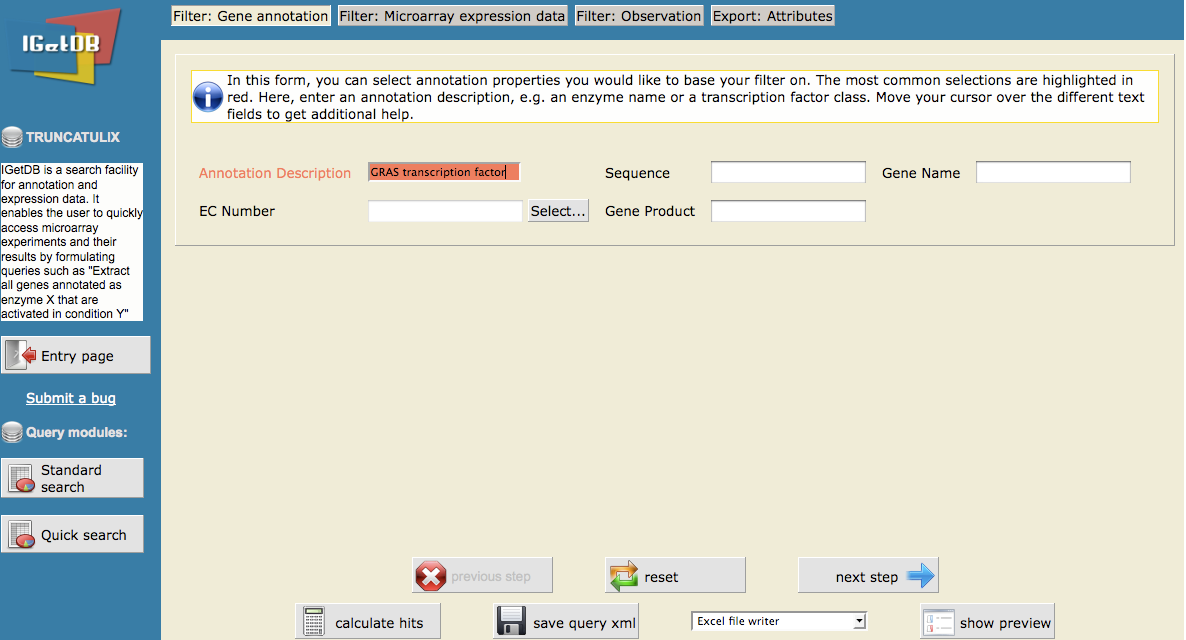
**The first filterstep – gene annotation**. The screenshot shows the filter page for the gene annotation data. The annotation descriptions can be queried, as well as the gene names, sequences and EC numbers.

To calculate this query, a user first specifies the standard search dialog on the left (see Figure 2). The user is guided through the different filter steps with an indicator bar on the top of the web pages. Help texts give examples and describe the options given by the different filters. Because some filters are more commonly used than others, the most commonly selected filters are highlighted in red.

For our example, the Annotation Description field is set to "GRAS transcription factor", due to our interest in GRAS genes. This way only genes that are annotated as GRAS transcription factor genes remain for the next filterstep.

**Figure 3:**
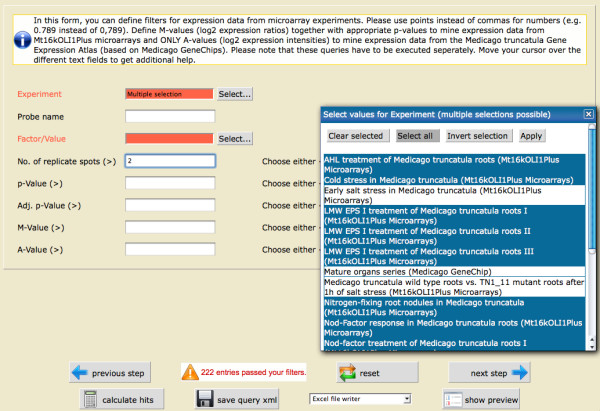
**The second filterstep – gene expression**. This screenshot shows the filter page for the expression data. The different integrated experiments can be selected, as well as different expression values and the number of replicates.

The next page directly informs the user that only 222 entries passed the first filterstep and asks for a filtering concerning microarray expression data. The user now selects all nodulation related experiments ("Nitrogen-fixing root nodules in *Medicago truncatula*", "Nod-factor response in *Medicago truncatula *roots", "Root endosymbiosis in *Medicago truncatula*", "AHL treatment of *Medicago truncatula *roots", "LMW EPS I treatment of *Medicago truncatula *roots I", "LMW EPS I treatment of *Medicago truncatula roots *II", "LMW EPS I treatment of *Medicago truncatula *roots III", "Nod-factor treatment of *Medicago truncatula *roots I", "Nod-factor treatment of *Medicago truncatula *roots II", "Response to phosphate in *Medicago truncatula *roots", "Nodulation development series, Mt oligo-dT primed") as Experiments and sets the No. of Replicates (>) to "2" (Figure 3).

In the next step, the user can specify results for the different functional annotation tools, as well as for KOG Categories and Gene Ontology numbers. They remain unused in our example, but more complex queries could use these to filter for specific KOG categories or GO numbers.

The last page shows the possible export options that can be selected for the remaining 35 entries. The previously selected filter criteria are preselected for the export.

**Figure 4:**
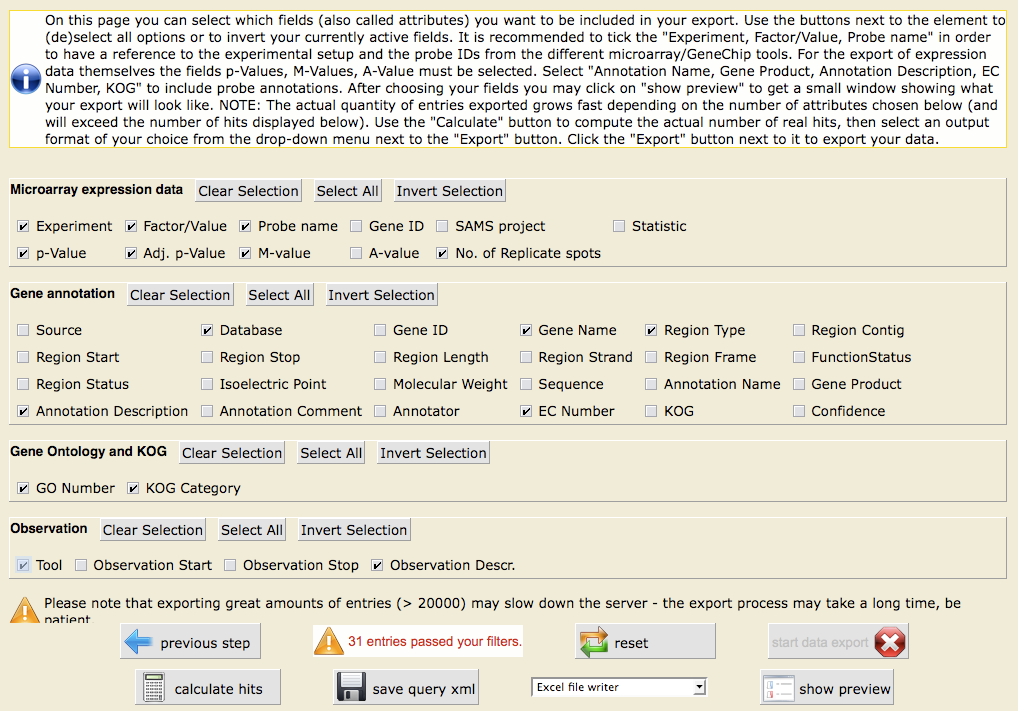
**Export options of TRUNCATULIX**. The screenshot shows the last page of the query dialog. The user can select which data and details should be exported, receiving an Excel, HTML, or csv file as result.

**Figure 5:**
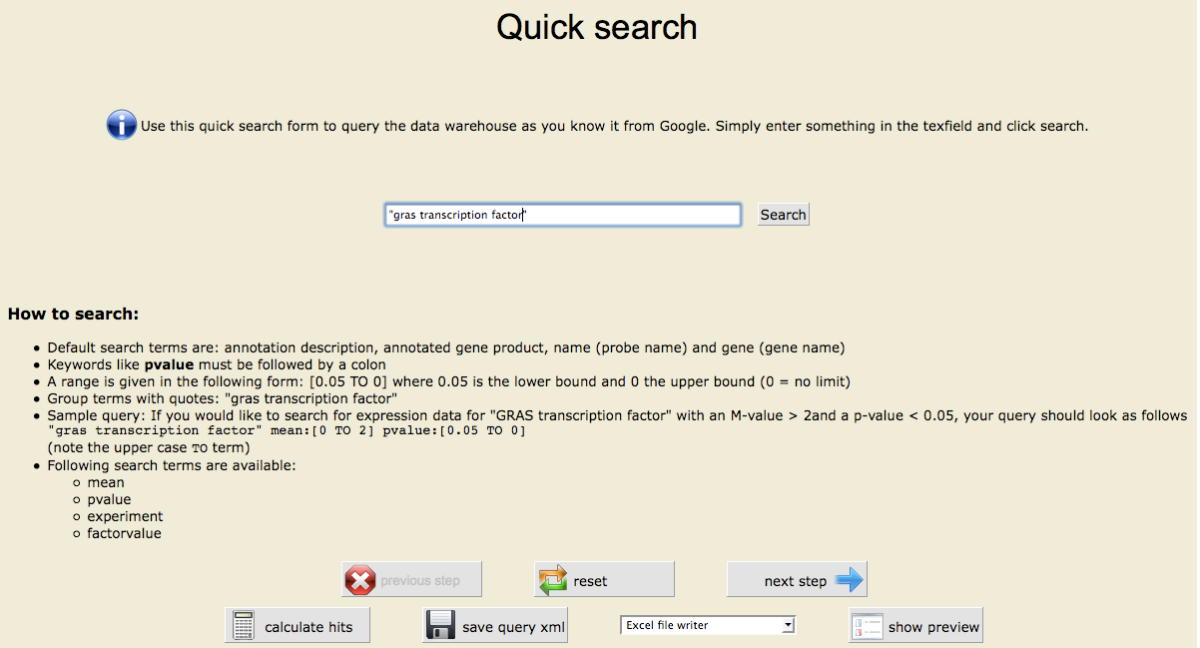
**The simple search dialog**. The screenshot shows the simple search dialog. The user can search for text fragments in the annotation of the genes, the gene names, the gene products, and the reporter names.

In this case, the export covers Database, Gene Name, Region Type, EC Number, and Annotation Description for the gene annotation; the Experiment name, Factor/Value, Probe name, p-Value, Adj. p-Value, M-Value, and no. of Replicates for the gene expression data from EMMA; the GO Number and the KOG Category, as well as the annotation Observations made by different Tools (Figure 4). Hitting the "calculate hits" button shows the number of datasets to be exported. This number may differ from the number of hits calculated previously based on sequence information because the new value is based on both sequence and expression information. To sneak a peek at the data, the user can use the preview option and browse through the first 100 results, or export the data as Excel, HTML, or csv file. A quick search – a more simple search interface found on the left side of the navigation panel – offers options to search for gene names, gene annotations, gene products, and repoter names using a google-like search interface (Figure 5).

After sumbitting the query, the details of the export can be selected as shown before using the complete query dialog (see Figure 4).

With the TRUNCATULIX data warehouse, it is now possible to have a look at the specific gene expressions for the different experiments and to find possibly interesting candidates for further experiments. Our example covers GRAS transcription factors. Some of them having already been reported to be key components of symbiotic signal transduction during nodulation [48,50,51].

The TRUNCATULIX data warehouse allows users easy access to sequencing, annotation, and expression research done at many laboratories worldwide.

## Discussion

Combining different data sources for fast searching and filtering is a widely used and common approach to overcome the immense manual work of searching for related data in every available database [52]. Related examples for this technique are **GeWare **[13] and the *Medicago truncatula ***Gene Expression Atlas **[46]. GeWare is a data warehouse that stores Affymetrix GeneChip^® ^microarray data combined with manually added annotations and data from public databases like GO, Ensembl, LocusLink and NetAffx [28,53-55]. The GeWare data warehouse focuses on the processing and analysis of microarrays, mostly containing clinical data. Various filter and export options are implemented.

The *Medicago truncatula *Gene Expression Atlas stores previously processed and analyzed gene expression and annotation data from Affymetrix GeneChip^® ^experiments. The data published is also available on ArrayExpress. The annotation data from Affymetrix is integrated into the data warehouse, but it can only be used for queries, it cannot be exported or viewed. In the same way, the GO numbers, KEGG functions, and the annotations of the genes can be queries. A homology search using Blast offers the opportunity to find genes according to their similarity to the Affymetrix GeneChip^® ^reporter or consensus sequences. The results of a query can be downloaded, but only the names of the reporters and the expression values are listed, the annotations are not shown and cannot be extracted.

In contrast to the other data warehouses, TRUNCATLIX not only stores the sequence and annotation data of the Affymetrix GeneChip arrays, but also sequence data from other genetic projects and institutes. The annotations that are provided are calculated using a well evaluated pipeline, offering KEGG, KOG, and GO numbers, if found in the annotations of homologues. TRUNCATULIX offers the option to export the results of a query, but in contrast to the the export of the *Medicago truncatula *Gene Expresison Atlas, not only the gene names and the expression values are exported, but (on request) also any other information that is integrated into the data warehouse. Table 4 compares the main features of the three data warehouses. The immense amount of intergrated sequence, annotation, and expression information makes the TRUNCATULIX data warehouse a very convenient resource in the field of *Medicago truncatula *research. TRUNCATULIX is freely available to the research community and additional expression data can be integrated upon request.

**Table 4:** A comparison of TRUNCATULIX to other data warehouses

Data Warehouse feature	GeWare	*Medicago truncatula *Gene Expression Atlas	TRUNCATULIX
Target organism	*homo sapiens*	*Medicago truncatula*	*Medicago truncatula*
static/dynamic data	dynamic	Static	static
number of microarray experiments	unknown	18	18
number of microarrays mircoarrays	unknown	54	204
automatic annotation information	yes	searchable, but not visible	yes
KEGG-mapping	no	yes	yes
GO-numbers	no	yes	yes
search	yes	yes	yes
blast homology search	no	yes	no, planned
export options	yes	yes	yes
free use	yes	yes	yes
free access	no	yes	yes

The table shows a comparison of the three data warhouses GeWare, the *Medicago truncatula *Gene Expression Atlas, and TRUNCATULIX.

### Future development

As more *Medicago trunculata *data becomes available from oligo-microarray and Affymetrix GeneChip^® ^experiments, it will be integrated into TRUNCATULIX. Additionally, a homology search using Blast will be implemetend.

## Conclusion

We created TRUNCATULIX, a data warehouse that combines data from microarray experiments with sequence data and high quality annotations in the area of *Medicago truncatula*. TRUNCATULIX is the first data warehouse in the field or *Medicago truncatula *research that offers the opportunity to search in all publicly available *Medicago truncatula *sequence data and expression data for different criteria and as a result to get a complete list of sequences, expression data, and annotations. Thus, a researcher can save much time and work finding interesting genes and results of previously conducted expression experiments. As the application uses an AJAX-based web interface, it can be used via a web browser and is platform independent. The results can be exported as Excel, HTML, or as csv files.

## Availability and requirements

The TRUNCATULIX data warehouse is freely available at .

## Authors' contributions

KH initialized the project, computed the annotations for the sequence data and is the main author of the manuscript. KR was responsible for the data schema, the data warehouse backend, and supervised the design of the web interface. TB helped on annotating the sequence data using SAMS. MD contributed in extracting of the microarray data from EMMA. TJ designed the web interface. HK analyzed some of the microarray data and supervised the export. AG supervised the integration of the different data sources. All authors revised and approved the final manuscript.
